# A novel inhibitory role of microRNA‐224 in particulate matter 2.5‐induced asthmatic mice by inhibiting TLR2

**DOI:** 10.1111/jcmm.14940

**Published:** 2020-01-24

**Authors:** Ping Li, Jinpeng Wang, Fengjun Guo, Baihong Zheng, Xuelei Zhang

**Affiliations:** ^1^ Department of Developmental Pediatrics The Second Hospital of Jilin University Changchun China; ^2^ Department of Cardiology The Second Hospital of Jilin University Changchun China; ^3^ Department of Gynaecology and Obstetrics The Second Hospital of Jilin University Changchun China; ^4^ Department of Pediatrics The Second Hospital of Jilin University Changchun China; ^5^ Key Laboratory of Wetland Ecology and Environment Northeast Institute of Geography and Agroecology Chinese Academy of Sciences Changchun China

**Keywords:** airway remodelling, asthma, microRNA‐224, particulate matter 2.5, TLR2/TLR4/MYD88 pathway, Treg/Th17

## Abstract

Epidemiological studies have shown that elevated concentrations of particulate matter 2.5 (PM2.5) correlate with increased incidence of asthma. Studies have highlighted the implication of microRNAs (miRNAs) in asthmatic response. Here, the objective of this study is to explore the effect of miR‐224 on PM2.5‐induced asthmatic mice. Ovalbumin (OVA) was utilized to establish asthmatic mouse models, which were then exposed to PM2.5, followed by miR‐224 expression detection. Next, lesions and collagen deposition area in lung tissue, ratio Treg/Th17, the expression of TLR4 and MYD88, inflammation, eosinophils (EOS) and airway remodelling were evaluated in OVA mice after injection with miR‐224 agomir. Following isolation of mouse primary bronchial epithelial cells, miR‐224 mimic and TLR2/TLR4 inhibitor were introduced to assess inflammation and the expression of TGF‐β, MMP9, TIMP‐1, Foxp3, RORγt, TLR2, TLR4 and MYD88. After exposure to PM2.5, lesions and collagen deposition were promoted in lung tissues, inflammation and EOS were increased in bronchoalveolar lavage fluid (BALF), and airway remodelling was enhanced in OVA mice. miR‐224 was down‐regulated, whereas TLR2/TLR4/MYD88 was up‐regulated in OVA mice after treatment with PM2.5, accompanied by Treg/Th17 immune imbalance. Of note, bioinformatic prediction and dual luciferase reporter gene assay confirmed that TLR2 was a target gene of miR‐224. Overexpressed miR‐224 reduced expression of TGF‐β, MMP9, TIMP‐1 and RORγt and inflammation but increased Foxp3 expression in bronchial epithelial cells through down‐regulating TLR2. In summary, overexpressed miR‐224 suppressed airway epithelial cell inflammation and airway remodelling in PM2.5‐induced asthmatic mice through decreasing TLR2 expression.

## 1 INTRODUCTION

Asthma is a complicated condition that gets recurrent episodes of airway obstruction in which airways narrow and produce extra mucus. Asthma is currently a chronic obstructive pulmonary disease and hard to be cured.^1^ There are approximately 10% of the people all over the world are affected by asthma, and this prevalence is still increasing in recent decades.^2^ The biological extrinsic risk factors of asthma include infections by viruses, bacteria and fungi. However, with the development of industry and the deteriorating environment, air pollution has become the main reason why asthma patients are increasing rapidly.^3^ Previous evidence has shown that tiny particulate matter (PM) is closely related to the pathogenicity and mortality rate of asthma.^4^, ^5^ For instance, heavy haze days that frequently occurred in the northern areas of China have been reported with significantly increased numbers of hospital admissions due to respiratory diseases, including acute bronchitis and asthma.^6^ A plenty of researches focus on the pathogenesis of asthma caused by microbes. However, studies on the pathogenesis of asthma caused by PM are limited. Therefore, it is urgent to investigate how air pollution affects the progression of asthma in order to reduce the risk by taking appropriate precautions.

Long‐term asthma always leads to allergic inflammation and airway remodelling. The allergens can induce epithelial cells to activate the secret interleukin directly or by specific binding to the Toll‐like receptor (TLR) on the membrane of dendritic cells, which then regulates the balance of T‐helper (Th) cells and T‐regulatory (Treg) cells through signalling pathway and generation of cytokines. Th2 cell is primarily verified to control the innate or allergic inflammation by secreting interleukin 4 and 13 (IL‐4 and IL‐13) with the help of Treg cell.^6^ Th17 cell is a new kind of T‐helper cell found recently and involved in autoimmune and inflammatory diseases.^6^ Furthermore, one study has suggested that Th17 cells can enhance inflammation in eosinophilic airway mediated by Th2‐cell.^7^ Although microbes' infection has been verified to activate Th2‐cell response via recognizing TLR2, but the underlying inflammatory mechanism involving particulate matter, especially PM2.5, in airway and lung tissue is still not well characterized. microRNA (miRNA) is a type of small non‐coding RNA that controls genes expression and further regulates cell cycle and cell differentiation.^8^ miRNAs have been demonstrated to participate in the regulation of asthma through controlling the differentiation and polarization of type 2 immune cells.^9^ Microarray technology has shown the altered expression pattern of miRNAs in asthma with down‐regulation of miR‐224.^10^ However, the specific roles of miR‐224 in the regulation of asthma progression are still unknown. Herein, we aim to investigate the role of miR‐224 in the airway remodelling and Treg/Th17 cells dynamics in ovalbumin (OVA)‐induced asthmatic mice affected by PM2.5. The present study proved that miR‐224 was an inhibitor of asthma progression aggravated by PM2.5.

## 2 METHODS

### 2.1 Ethics statement

All experiments involving animals in this study were approved by the Experimental Ethics Committee of the Second Hospital of Jilin University (NO. 235). The animal experiments were strictly designed and executed according to the Guide for the Care and Use of Laboratory Animal by International Committees. All efforts were made to minimize the number of animals used in the experiments and their suffering.

### 2.2 PM2.5 sampling and processing

The atmospheric PM2.5 samples were collected by Thermo Scientific and detected by TEOM1405‐D particle monitor. The PM2.5 was then collected onto filters, and the total mass of PM2.5 was measured using a Tapered Element Oscillating Microbalance. Before being weighed, the glass fibre membrane of atmospheric PM2.5 was oven‐dried at 60°C for 6 hours. The sampler was applied with the negative pressures of −1.9 to −2.1 kPa for the collection of PM2.5. The membrane was replaced with a new one when the negative pressure reached −2.2 kPa, and filters were dried for 24 hours at 60°C, followed by being weighed. Filters containing PM2.5 were cut into pieces (2 × 1 cm), pooled into a 100 mL deionized distilled water and treated using ultrasonic sonication for 2 hours. The liquid containing the particulate matter was filtered through six layers of sterile gauze and centrifugated at 1800 *g* for 30 minutes at 4°C. After being vacuum‐freeze dried and weighed, PM2.5 was suspended in sterile saline to obtain PM2.5 suspensions (1.6 mg/mL) for the establishment of mouse model. The suspension was then stored at −20°C.^11^

### 2.3 Study subjects

Fifty female BALB/c mice aged 4‐8 weeks and weighing 20 ± 2 g were obtained from Changchun Yisi Laboratory Animal Technology Co., Ltd. (the certification No SCXK(Ji) 2018‐0007). The mice were allowed to adapt to the environment of laboratory conditions (temperature: 24 ± 1°C; relative humidity: 50%‐70%) and adaptively fed for 1 week with free access to food and water.^12^

### 2.4 Model establishment and grouping

Ten mice were selected as normal controls, another ten mice were treated with only ovalbumin (OVA) (nine mice, success rate: 90%), and another ten mice were treated with PM2.5 and OVA (eight mice, success rate: 80%). Eight mice of three treatment groups were taken for subsequent experiments. The mice with OVA or PM2.5 and OVA were injected subcutaneously and intraperitoneally with 0.5 mL sensitizing solution supplemented with 25 μg OVA (A‐5253, Sigma‐Aldrich Corp.) and 2 mg aluminium hydroxide gel (AL(OH)_3_) (V‐900163, Sigma‐Aldrich Corp.) on day 1 and day 8, respectively. Then, these mice were stimulated with 5% OVA for 30 minutes every day from day 15 to day 28 and every other day from day 30 to day 42. Normal control mice and OVA mice were injected with 40 μL normal saline (NS), whereas mice with PM2.5 and OVA were injected with 40 μL PM2.5 solution (1.6 mg·kg^−1^) through trachea on days 29, 33, 37 and 41, respectively. After the last treatment for 24 hours, the mice were killed under aseptic conditions, followed by sample collection. The remaining 20 mice were used for mouse model with the above procedures (success rate: 95%), which received an injection with NC agomir or miR‐224 agomir after PM2.5 and OVA treatment (n = 10). The rest of the processing was the same as stated above.^12^

### 2.5 Culture of mouse primary bronchial epithelial cells

After the mice were killed under sterile conditions, the bronchi were collected and immediately placed into pre‐cooled Dulbecco's modified Eagle medium (DMEM)/F12 containing streptomycin and transferred into phosphate buffer saline (PBS) to remove the surrounding connective tissue and blood vessels and cut longitudinally. The cells were detached with the medium that consisted of 1:1 mixture of DMEM/F12 containing streptomycin supplemented with pronase solution for 24 hours at 4°C, followed by addition with DMEM/F12 medium containing streptomycin and foetal bovine serum (FBS). After that, the tube was gently inverted 12 times and then repeated once. After centrifugation, the cells were resuspended in DMEM/F12 medium containing streptomycin and 10% FBS. The cell suspension was seeded into a Petri dish, whereas the unattached cells were collected 2 hours later. The cells were seeded into a 24‐well plate coated with collagen and cultured in DMEM/F12 medium containing streptomycin and FBS at 37°C with 5% CO_2_ for 24 hours. The next day, the DMEM/F12 medium containing streptomycin and FBS was discarded. Subsequently, the cells were added with DMEM/F12 medium containing growth factors and cultured at 37°C with 5% CO_2_. Mimic NC, miR‐224 mimic, dimethylsulphoxide (DMSO) and GY03865 (TLR2/TLR4 inhibitor, Hangzhou Guangyuan Biotechnology Co., Ltd.) were then introduced into the cells. The mimic NC and miR‐224 mimic were purchased from GenePharma.^13^

### 2.6 Haematoxylin‐eosin (HE) staining

The lung tissues of mice were fixed in 4% paraformaldehyde for 24 hours; dehydrated by 80%, 90% and 100% ethanol and n‐butanol; and then placed in a wax box at 60°C for waxing. The tissues were then embedded in paraffin and cut into 5‐μm‐thick sections. The sections were then baked at 60°C for 1 hour and dewaxed with xylene. After hydration, HE staining was performed and the level of lung inflammation was evaluated by inflammatory cell infiltration around the bronchial tubes as follows: 0, no; 1, mild; 2, moderate; 3, significant; and 4, severe. Briefly, the prepared sections were dewaxed, hydrated with gradient alcohol, stained with haematoxylin (Beijing Solarbio Science & Technology Co., Ltd.) for 2 minutes, washed with tap water for 10 seconds and then differentiated by 1% hydrochloric acid for 10 seconds. After 1 minute of wash with distilled water, the sections were stained with eosin solution for 1 minute, washed with distilled water for 10 seconds, dehydrated with gradient alcohol, cleared with xylene and then sealed with neutral gum. Thereafter, the morphological changes of lung tissue were observed under an optical microscope (XP‐330, Shanghai Bingyu Optical Instrument Co., Ltd.). Three small and medium bronchi with complete cross‐section were selected in each mouse with 200 times magnification under a light microscope using Image‐Proplus 6.0 medical image analysis software. The total bronchial wall area (WAt), smooth muscle area (WAm) and the number of nuclei in ASMC (N) were measured, expressed as the area of the basement membrane per unit length and represented by WAt, WAm and N, respectively.

### 2.7 Masson's trichrome staining

The lung tissues of mice were fixed in 4% paraformaldehyde for 24 hours; dehydrated by 80%, 90% and 100% ethanol and n‐butanol; immersed in a 60°C wax box; embedded; sectioned; and dewaxed. After hydration, the tissues were stained with Regaud haematoxylin staining solution for 5‐10 minutes and washed with distilled water thoroughly. After washing, the tissues were stained with Masson Ponceau red acidic fuchsin solution for 5‐10 minutes, then immersed in 2% glacial acetic acid aqueous solution and differentiated with 1% phosphomolybdic acid aqueous solution for 3‐5 minutes. Without washing with water, the tissues were dyed with aniline blue or light green solution for 5 minutes and then immersed in 0.2% aqueous glacial acetic acid solution. The tissues were dehydrated with gradient alcohol, followed by clearing with xylene and blocking with neutral gum. Finally, the tissues were observed under a microscope and the collagen deposition area was quantitatively analysed using NIH Image J image analysis software.

### 2.8 Enzyme‐linked immunosorbent assay (ELISA)

The bronchoalveolar lavage (BAL) was performed three times using 1.2 mL PBS. The lavage fluid was collected in a 4°C low‐temperature centrifuge, followed by centrifugation at 450 *g* for 10 minutes. The supernatant was taken as a sample at −20°C for subsequent experiments. The levels of IL‐4 (JLC3599), IL‐5 (JLC3600), IL‐6 (JLC3601), IL‐10 (JLC3565) and IL‐13 (JLC3556) were detected according to the manufacturer's instructions of ELISA kit (Shanghai Jing Kang Biotechnology Co., Ltd. The cell culture medium of each well was collected and centrifuged at 225 *g* for 10 minutes, and the cell supernatant was taken. The levels of IL‐4, IL‐5, IL‐10 and IL‐17 were detected in strict accordance with the manufacturer's instructions of the ELISA kit.

### 2.9 Determination of eosinophils (EOS) ratio

The bronchoalveolar lavage fluid (BALF) was centrifuged at 225 *g* for 10 minutes, and the supernatant was removed. The cell precipitation was resuspended to prepare a smear for staining. The percentage of EOS in total white blood cells was calculated by cell counting under an optical microscope.

### 2.10 Isolation of mRNA and reverse transcription quantitative polymerase chain reaction (RT‐qPCR)

Isolation of total RNA from the cells was performed using Trizol (15596026, Invitrogen) and was reversely transcribed into complementary DNA (cDNA) using PrimeScript RT reagent kit (RR047A) or NCode miRNA First‐Strand cDNA Synthesis Kit (Thermo Fisher Scientific Inc). The synthesized cDNA was used to perform RT‐qPCR reactions using Fast SYBR Green PCR Kit (Applied Biosystems) on ABI PRISM 7300 RT‐PCR System (Applied Biosystems). The U6 gene was used as an endogenous control gene for normalizing the expression of miR‐224, whereas glyceraldehyde‐3‐phosphate dehydrogenase (GAPDH) was taken as the internal reference of other genes. The fold changes between the experiment group and the control group were calculated by means of relative quantification (2^−ΔΔCt^ method). Primer sequences are shown in Table 1.^14^, ^15^

**Table 1:** Primer sequences for RT‐qPCR

Genes	Primer sequences
miR‐224	F: 5′‐CTGGTAGGTAAGTCACTA‐3′
	R: 5′‐TCAACTGGTGTCGTGGAG‐3′
Foxp3	F: 5′‐CACCCAGGAAAGACAGCAACC‐3′
	R: 5′‐GCAAGAGCTCTTGTCCATTGA‐3′
RORγt	F: 5′‐AGTGTAATGTGGCCTACTCCT‐3′
	R: 5′‐GCTGCTGTTGCAGTTGTTTCT‐3′
U6	F: 5′‐AACGCTTCACGAATTTGCGT‐3′
	R: 5′‐CTCGCTTCGGCAGCACA‐3′
GAPDH	F: 5′‐ACCACAGTCCAT GCCATCAC‐3′
	R: 5′‐TCCACCACCCTGTTGCTGTA‐3′

Abbreviations: F, forward; GAPDH, glyceraldehyde‐3‐phosphate dehydrogenase; R, reverse; RT‐qPCR, reverse transcription quantitative polymerase chain reaction.

### 2.11 Western blot analysis

Total protein was extracted using radioimmunoprecipitation assay (RIPA) lysis buffer (Boster Biological Technology Co. Ltd.) containing protease inhibitors. The protein concentration was determined using a bicinchoninic acid (BCA) protein assay kit (Boster Biological Technology Co. Ltd.). Next, the protein was separated by 10% sodium dodecyl sulphate‐polyacrylamide gel electrophoresis (SDS‐PAGE) and transferred onto the poly(vinylidene fluoride) membranes. The membrane was then blocked with 5% bovine serum albumin (BSA) at room temperature for 2 hours to avoid non‐specific binding, followed by overnight incubation with the following primary rabbit polyclonal antibodies (Abcam Inc) to TLR2 (ab213676, 1:500), TLR4 (ab13556, 1:500), MYD88 (ab2064, 1:1000), TGF‐β (ab92486, 1:500), MMP9 (ab38898, 1:500), TIMP‐1 (ab61224, 1:1000) and GAPDH (ab181602, 1:5000) at 4°C. Afterwards, horseradish peroxidase‐conjugated secondary antibody goat anti‐mouse (ab205719, 1:2000, Abcam Inc) was added to the membrane and incubated for 1 hour at room temperature. The membrane was visualized using an enhanced chemiluminescence (ECL) kit (EMD Millipore). The greyscale quantification of bands in Western blot images was performed using Image J analysis software with GAPDH as an internal reference.^16^

### 2.12 Flow cytometry

The lung tissue was employed to prepare single‐cell suspension and centrifuged at 375 *g* for 5 minutes, with the supernatant discarded. The tissues were added with 2 mL of red blood cell lysate and cultured for 8 minutes at room temperature. The supernatant was discarded by centrifugation after two PBS washes. The cells were made into single‐cell suspension using Roswell Park Memorial Institute (RPMI) 1640 culture medium and added with 50 ng/mL phorbol myristate acetate (PMA) (Sigma‐Aldrich Corp.), 500 ng/mL ionomycin (Sigma‐Aldrich Corp.) and 0.7 μL/mL GolgiPlug (BD Biosciences) for 4‐6 hours incubation at 37°C with 5% CO_2_. The cells were centrifuged to remove the supernatant, mixed completely and added with fluorescein isothiocyanate (FITC)‐conjugated anti‐mouse CD4 antibodies (Cat #11‐0441‐82) for T1, T2 and T17 analysis. Cells were incubated with FITC‐conjugated cells containing anti‐mouse CD4 and phenyleneethynylene (PE)‐conjugated anti‐mouse CD25 (Cat #12‐0251‐82) for Treg analysis. After surface staining, the cells were resuspended. Anti‐mouse Foxp3 APC (Cat #17‐5773‐82)/anti‐mouse IL‐17 APC (Cat #17‐7177‐81) was then stained in a solidified and permeabilized solution according to the manufacturer's instructions. All antibodies from eBioscience were detected using flow cytometer.^11^

### 2.13 Bioinformatics website and dual luciferase reporter gene assay

The target gene analysis of miR‐224 and TLR2 was performed using a biological prediction site miRmap. The cells were seeded into a 6‐well plate at a density of 2 × 10^5^ per well. After the cells were attached to the wells, transfection was carried out according to the above method. After successful transfection, the cells were incubated for 48 hours and then collected. The luciferase activity of miR‐224 and TLR2 in cells was measured according to the manufacturer's instructions in Genecopoeia's dual luciferase assay kit (D0010, Beijing Solarbio Science & Technology Co., Ltd.). Fluorescence intensity was measured using a Promega Glomax 20/20 luminometer fluorescence detector (E5311, Shaanxi Zhongmei Biotechnology Co., Ltd.).

### 2.14 Statistical analysis

Statistical analysis was conducted by SPSS 21.0 statistical software (IBM Corp.). Measurement data were expressed as mean ± SD. The data confirming to normal distribution and homogeneity of variance between two groups with the unpaired design were compared by unpaired *t* test. Data among multiple groups were analysed by one‐way analysis of variance (ANOVA), followed by Tukey's post hoc test. A *P* value of <.05 was considered statistically significant.

## 3 RESULTS

### 3.1 PM2.5 induced inflammation and airway remodelling in asthmatic mice

**Figure 1:**
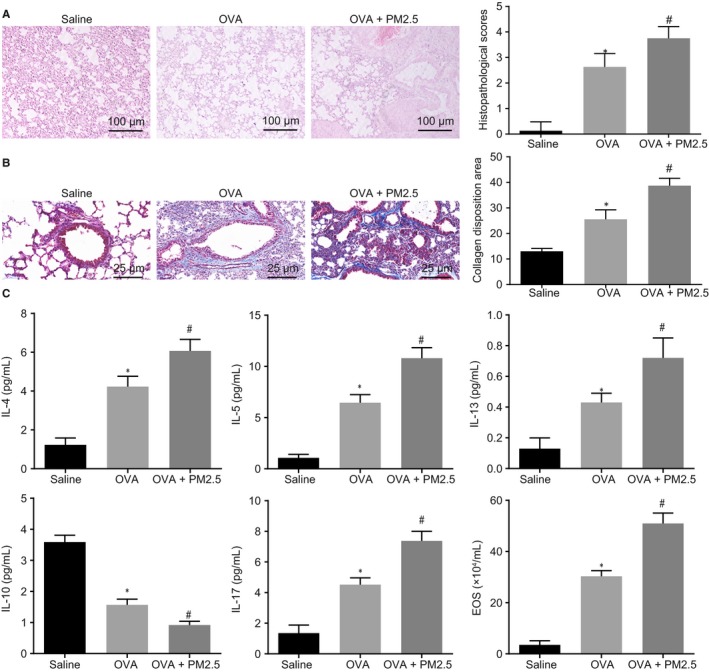
PM2.5 induced the inflammation and airway remodelling in asthmatic mice. The mice were treated with saline, OVA and OVA + PM2.5. A, The lung tissue lesions in mice detected by HE staining (100×). B, The collagen deposition area in mice lung tissue detected by Masson's trichrome staining (400×). C, The expression of inflammatory factors and EOS in BALF of mice. *P < .05 compared with saline‐treated mice; ^#^ *P* < .05 compared with OVA‐treated mice. The measurement data from three independent experiments were expressed as mean ± SD. Differences between multiple groups were analysed by one‐way ANOVA, followed by Tukey's post hoc test. n = 8

Firstly, the asthmatic mouse model was established and their behaviour was observed. The normal eating habits and behaviour, as well as regular respiratory rhythm without abnormal symptom, were observed in saline‐treated mice. On the other hand, the OVA‐treated mice showed obvious irritability, shortness of breath, snoring, coughing, slight tremor of the limbs, bow back and decrease in mobility, with mild cyanosis in nose and mouth, as well as nodding breathing occurring in later period, which also was observed in the mice treated with OVA + PM2.5. The mild cyanosis in nose and mouth, as well as nodding breathing, has been observed earlier in mice treated with OVA + PM2.5 than the OVA‐treated mice, indicating that the OVA‐induced asthmatic mouse model was successfully established. The lung tissue lesions of mice were detected by HE staining. The results showed that the inflammatory cell infiltration of the lung tissue and histopathological score were increased in OVA‐treated mice, which was further elevated by treatment with PM2.5 (Figure 1A). The collagen deposition area was detected by Masson's trichrome staining which displayed that the area of collagen deposition was increased after OVA treatment, which was further promoted by PM2.5 (Figure 1B). The expression of inflammatory factors in BALF and EOS was detected. It was found that the expression of IL‐4, IL‐5, IL‐13 and IL‐17, as well as EOS, was increased in BALF of the OVA‐treated mice, whereas IL‐10 showed a decrease in expression. The expression of IL‐4, IL‐5, IL‐13 and IL‐17 and EOS in BALF was significantly increased in mice treated with OVA + PM2.5 vs the OVA‐induced mice, whereas IL‐10 showed a significant decrease in expression (*P* < .05) (Figure 1C). Hematoxylin‐eosin staining was used to detect the bronchial indexes, and the results are shown in Table 2. The WAt, WAm and N were increased after OVA treatment, which was further increased after co‐treatment with PM2.5. Based on the above evidence, OVA successfully stimulated the establishment of the asthmatic mouse model, and PM2.5 induced asthma exacerbation in mice with increased inflammation and airway remodelling.

**Table 2:** Indexes showing mouse bronchial conditions in response to OVA and PM2.5

Group	Wat/Pbm (μm^2^/μm)	Wam/Pbm (μm^2^/μm)	N/Pbm (number/μm)
Saline	5.83 ± 0.74	3.22 ± 0.63	0.02 ± 0.01
OVA	10.29 ± 0.98	6.81 ± 0.84	0.05 ± 0.03
OVA + PM2.5	16.47 ± 2.19	8.43 ± 1.32	0.07 ± 0.03

Note

The measurement data from three independent experiments were expressed as mean ± SD. Differences of multiple groups were analysed by one‐way ANOVA, followed by Tukey's post hoc test. n = 8.

* *P* < .05 compared with saline‐treated mice.

^#^ *P* < .05 compared with OVA‐treated mice.

### 3.2 PM2.5 activated TLR2/TLR4/MYD88 pathway to induce Treg/Th17 immune imbalance

**Figure 2:**
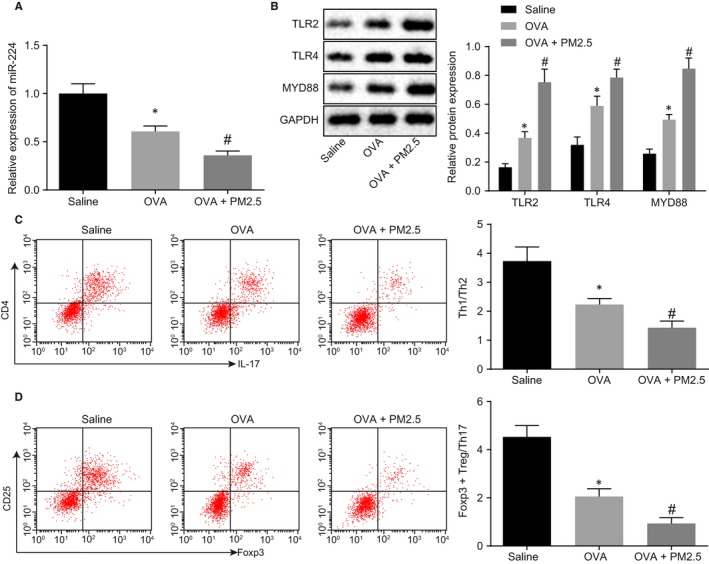
PM2.5 induced Treg/Th17 immune imbalance by activation of the TLR2/TLR4/MYD88 pathway. The mice were treated with saline, OVA and OVA + PM2.5. A, miR‐224 expression in mice measured by RT‐qPCR. B, Western blot analysis of TLR2/TLR4/MYD88 protein in mice. C, The ratio of Th1 and Th2 cells in lung tissue detected by flow cytometry. D, The ratio of Foxp3+ Treg and Th17 cells in lung tissue was detected by flow cytometry. *P < .05 compared with saline‐treated mice; ^#^ *P* < .05 compared with OVA‐treated mice. The measurement data from three independent experiments were expressed as mean ± SD. Differences between multiple groups were analysed by one‐way ANOVA, followed by Tukey's post hoc test. n = 8

In order to explore the effect of PM2.5 on miR‐224 and TLR2/TLR4/MYD88 pathway, miR‐224, TLR2, TLR4 and MYD88 expression was assessed in the lung tissue of model mice. As depicted in Figure 2A, the expression of miR‐224 was decreased in the OVA‐treated mice and was further reduced in mice treated with OVA + PM2.5. In addition, after mice were treated with OVA, the expression of TLR2, TLR4 and MYD88 was increased. The expression of TLR2, TLR4 and MYD88 was significantly higher in mice treated with OVA + PM2.5 than in OVA‐treated mice (*P* < .05; Figure 2B). The expression of Th2 cells, Th17 cells and Treg cells in lung tissue of mice was detected by flow cytometry. The results revealed that after OVA treatment, Th2 was increased in CD4+ T cells, whereas the percentage of Th17 cells was increased, the percentage of Foxp3+ Treg cells and Th1 cells were significantly decreased, and the percentage of Th1/Th2 and Foxp3+ Treg/Th17 were profoundly decreased. These results were similar by comparing mice treated with OVA + PM2.5 to OVA‐treated mice (*P* < .05; Figure 2C,D). The contents of IFN‐γ, IL‐17 and IL‐10 in serum are shown in Table 3.  When mice were solely treated with OVA, the levels of IFN‐γ and IL‐10 in serum were decreased, but that of IL‐17 was increased, which was exacerbated by further treatment with PM2.5. The above results demonstrated that miR‐224 was down‐regulated in PM2.5‐treated asthmatic mice, whereas the expression of TLR2, TLR4 and MYD88, as well as Treg/Th17 immune imbalance, was increased in PM2.5‐treated asthmatic mice.

**Table 3:** The content of IFN‐γ, IL‐17 and IL‐10 in serum of peripheral blood detected by ELISA

Group	IFN‐γ (pg/mL)	IL‐17 (pg/mL)	IL‐10 (pg/mL)
Saline	117.12 ± 21.01	179.48 ± 25.09	103.18 ± 13.74
OVA	53.25 ± 5.83*	215.11 ± 27.81*	80.98 ± 9.96*
OVA + PM2.5	26.35 ± 3.54#	258.14 ± 29.42#	41.25 ± 6.31#

Note

The measurement data from three independent experiments were expressed as mean ± SD. Differences of multiple groups were analysed by one‐way ANOVA, followed by Tukey's post hoc test. n = 8.

* *P* < .05 compared with saline‐treated mice.

^#^ *P* < .05 compared with OVA‐treated mice.

### 3.3 miR‐224 directly targets TLR2 and down‐regulates the expression of TLR2

**Figure 3:**
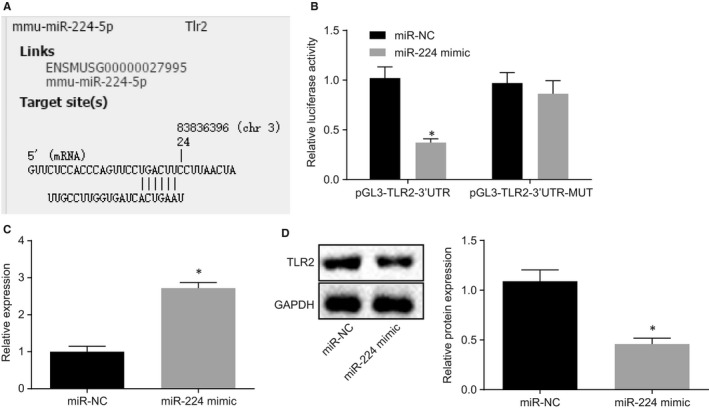
TLR2 was a target gene of miR‐224. A, The predicted binding sites between miR‐224 and TLR2 in miRmap database. B, The luciferase activity detected by dual luciferase reporter gene assay. C, The expression of miR‐224 after miR‐224 mimic treatment detected by RT‐qPCR. D, The expression of TLR2 after miR‐224 mimic treatment detected by Western blot analysis. *P < .05 compared with cells introduced with mimic NC. The measurement data from three independent experiments were expressed as mean ± SD. Data confirming to normal distribution and homogeneity of variance between two groups with the unpaired design were compared by unpaired *t* test. The experiment was repeated 3 times

Based on the miRmap database predictive analysis, there was a specific binding region between TLR2 gene sequence and miR‐224 sequence, and TLR2 was the target gene of miR‐224 (Figure 3A), which was verified by luciferase reporter gene assay in HEK‐293T cells. As shown in Figure 3B, the luciferase activity of the wild‐type pGL3‐TLR2‐3′untranslated region (3′UTR) was decreased by treatment with miR‐224 mimic, whereas there was no significant difference of the luciferase activity of the mutant pGL3‐TLR2‐3′UTR (*P* > .05). Then, miR‐224 was up‐regulated in the mouse primary bronchial epithelial cells, and the expression of miR‐224 and TLR2 in cells was evaluated. As described in Figure 3C,D, the expression of miR‐224 was increased but TRL2 expression was reduced after treated with miR‐224 mimic. The above results demonstrated that TLR2 was a target gene of miR‐224 and was down‐regulated by miR‐224.

### 3.4 Overexpression of miR‐224 down‐regulates TLR2 to inhibit inflammation and reduce the expression of MMP9 and TIMP‐1 in bronchial epithelial cells

**Figure 4:**
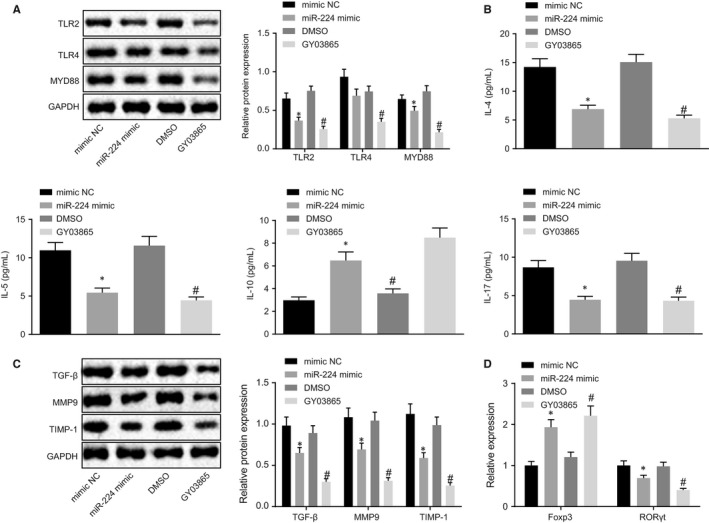
Bronchial epithelial cell inflammation was suppressed by down‐regulation of miR‐224–mediated TLR2. Bronchial epithelial cells were treated with mimic NC, miR‐224 mimic, DMSO and GY03865. A, The levels of TLR2, TLR4 and MYD88 detected by Western blot analysis. B, The expression of inflammatory factors detected by ELISA. C, The expression of TGF‐β, MMP9 and TIMP‐1 detected by Western blot analysis. D, The expression of Foxp3 and RORγt detected by RT‐qPCR. *P < .05 compared with cells treated with mimic NC; ^#^ *P* < .05 compared with cells treated with GY03865. The measurement data from three independent experiments were expressed as mean ± SD. Differences between multiple groups were analysed by one‐way ANOVA, followed by Tukey's post hoc test. The experiment was repeated 3 times

Bronchial epithelial cells were treated with miR‐224 and TLR2/TLR4 inhibitor to investigate the effects of miR‐224 and TLR2/TLR4 on bronchial epithelial cells. Western blot analysis described that when miR‐224 was overexpressed in bronchial epithelial cells, the expression of TLR2 and MYD88 was reduced, and there was no significant difference in TLR4 expression (*P* > .05). Moreover, the expression of TLR2, TLR4 and MYD88 was decreased by treatment with TLR2/TLR4 inhibitor (Figure 4A). Next, the expression of inflammatory cytokines was determined by ELISA. The results indicated that the expression of IL‐4, IL‐5 and IL‐17 was obviously reduced when treated with miR‐224 mimic or TLR2/TLR4 inhibition, whereas the expression of IL‐10 was significantly increased (Figure 4B). Western blot analysis was performed to identify the levels of TGF‐β, MMP9 and TIMP‐1 in cells. The levels of TGF‐β, MMP9 and TIMP‐1 were reduced following ectopic expression of miR‐224 or suppression of TLR2/TLR4 (Figure 4C). The expression of Foxp3 and RORγt was detected by RT‐qPCR, as shown in Figure 4D. Up‐regulation of miR‐224 or inhibition of TLR2/TLR4 resulted in an increase in Foxp3 expression but decrease in RORγt expression. Based on the above results, the overexpression of miR‐224 inhibited TLR2 expression, thereby suppressing the bronchial epithelial cell inflammation.

### 3.5 Overexpression of miR‐224 down‐regulates TLR2 expression and alleviates inflammation and airway remodelling in PM2.5‐induced asthmatic mice

**Figure 5:**
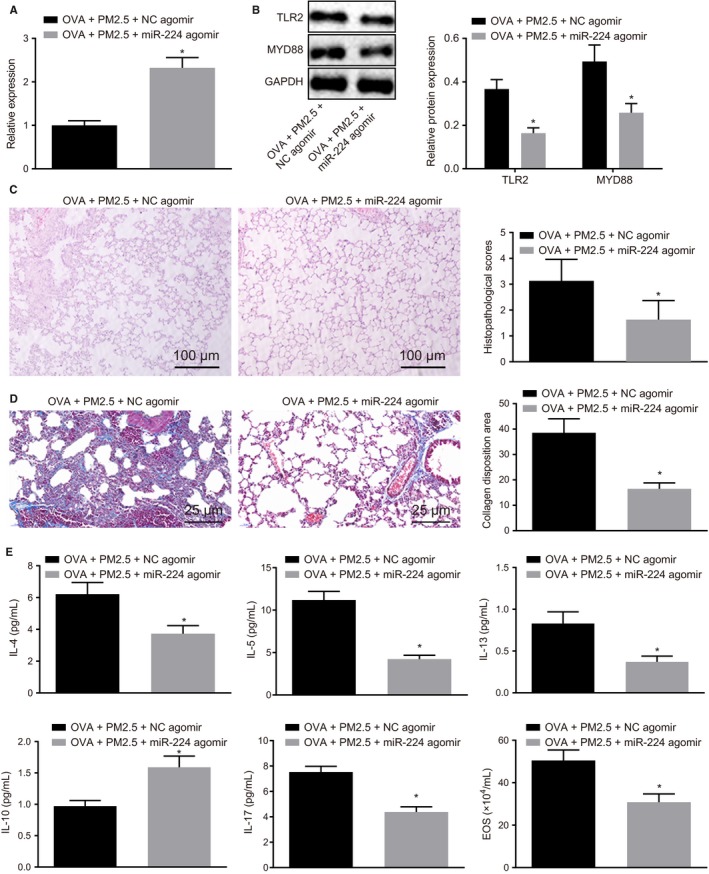
Overexpression of miR‐224 results in inhibition of TLR2 expression and suppression of asthma progression in vivo. Mice were treated with OVA + PM2.5 + miR‐224 agomir or OVA + PM2.5 + NC agomir. A, The expression of miR‐224 detected by RT‐qPCR. B, The expression of TLR2 and MYD88 detected by Western blot analysis. C, Lung tissue lesions of mice detected by HE staining (100×). D, The collagen deposition area in the lung tissue of mice detected by Masson's trichrome staining (400×). E, The expression of inflammatory factors in BALF and EOS detected by ELISA and eosinophil ratio assay. *P < .05 compared with mice treated with OVA + PM2.5 + NC agomir. The measurement data from three independent experiments were expressed as mean ± SD. Data confirming to normal distribution and homogeneity of variance between two groups with the unpaired design were compared by unpaired *t* test. n = 8

After treatment with OVA and PM2.5, mice were injected with miR‐224 agomir to explore the effect of miR‐224 on inflammation and airway remodelling in mice. Results demonstrated that increased expression of miR‐224 (Figure 5A) and the reduced expressions of TLR2 and MYD88 were observed in PM2.5‐treated OVA mice after injection of miR‐224 agomir (Figure 5B). Subsequently, HE staining was performed to detect lung tissue lesions in mice. As shown in Figure 5C, the inflammatory cell infiltration and histopathological score were significantly reduced in the lung tissue of mice by miR‐224 up‐regulation in the presence of OVA and PM2.5. The collagen deposition area was measured by Masson's trichrome staining. The results documented that after treated with OVA and PM2.5, ectopic expression of miR‐224 has led to the reduction of the collagen deposition area (Figure 5D). The expression of inflammatory factors and EOS in BALF was detected by ELISA and eosinophil ratio assay. The results showed that IL‐4, IL‐5, IL‐13 and IL‐17 expression, as well as EOS in BALF, was significantly reduced, whereas IL‐10 expression was significantly increased after miR‐224 was up‐regulated in mice treated with OVA and PM2.5 (Figure 5E). The bronchial indexes were detected by HE staining, and results were documented in Table 4. Following ectopic expression of miR‐224, WAt, WAm and N was significantly reduced in the mice treated with OVA and PM2.5. The above results demonstrated that the overexpression of miR‐224 could reduce PM2.5‐induced inflammation in asthmatic mice and airway remodelling.

**Table 4:** Mouse bronchial related index

Group	Wat/Pbm (μm^2^/μm)	Wam/Pbm (μm^2^/μm)	N/Pbm (number/μm)
OVA + PM2.5 + NC agomir	16.47 ± 2.19	9.48 ± 1.32	0.09 ± 0.02
OVA + PM2.5 + miR‐224 agomir	13.83 ± 0.74	3.22 ± 0.63	0.05 ± 0.03

Note

The measurement data from three independent experiments were expressed as mean ± SD. Confirming to normal distribution and homogeneity of variance, the data of two groups with unpaired design were compared by unpaired *t* test. n = 8.

*P < .05 compared with mice treated with OVA + PM2.5 + NC Agomir.

## 4 DISCUSSION

**Figure 6:**
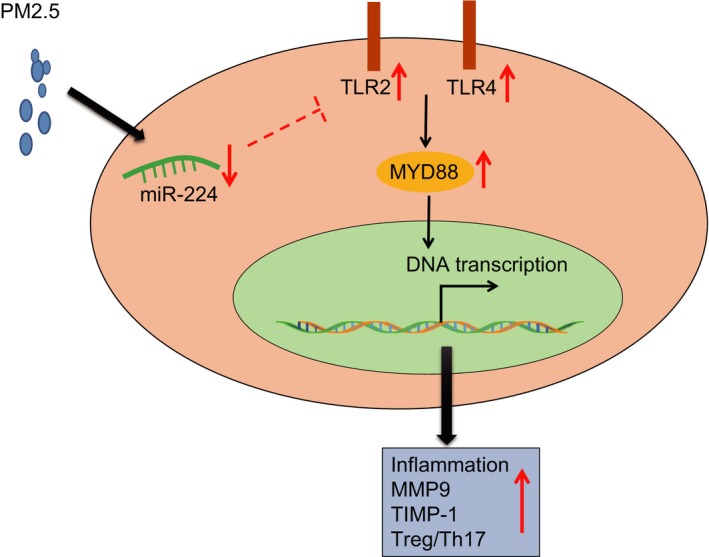
PM2.5 treatment activates TLR2/TLR4/MYD88, which acts to affect inflammation and airway remodelling in OVA‐induced asthmatic mice. Overexpression of miR‐224 inhibits airway epithelial cell inflammation and alleviates airway remodelling in mice by down‐regulating TLR2 expression

The occurrence of asthma is rising among people across the world annually under exasperating air pollution. Asthma can affect patients with various symptoms including continuous coughing, shortness of breath and tightness or pain in the chest, which leads to struggling lifestyle and heavy medical cost on the drugs to alleviate the syndromes.^17^ Therefore, the development of medical treatment is critically needed to improve the current prevention and treatment of patients with asthma. In this study, we found that PM2.5 could aggravate inflammatory response and airway remodelling, and induce Treg/Th17 cell imbalance via activation of the TLR2/TLR4/MYD88 pathway (Figure 6). The present study showed that overexpression of miR‐224 could inhibit the inflammation caused by PM2.5 by targeting TLR2 specifically, which suggested that miR‐224 may act as an inhibitor against the development of asthma.

First of all, PM2.5 could increase the secretion of several interleukins and break the immune balance of Treg/Th17 cells, which eventually exacerbated the inflammation in lung tissue of OVA mouse model. PM2.5 is a fine solid particle with diameter no more than 2.5 µm; therefore, it can enter and invade the smallest airways of the respiratory system. PM2.5 has a large surface‐volume ratio and is able to absorb plenty of toxic molecules and microbes that are primarily thought to be the only allergic antigen to human.^18^, ^19^ The PM2.5 samples that were used in the present study were strictly sterilized and applied to the contact tubes in the lung of OVA mice. Results showed that increased lung inflammation and reduced airway remodelling were observed in mice, which indicated that PM2.5 may act as an allergic antigen to activate the inflammatory response. The potential role of PM2.5 as an allergic antigen has been validated by previous studies. For instance, several interleukin receptors, such as IL‐1R1 and IL‐6R, have been shown to be up‐regulated in human bronchial epithelial cells (BEAS‐2B) when cultured with PM2.5.^20^ Besides, rats that exposed to air containing PM2.5 in short term were examined with inflammatory cell infiltration and lung tissue congestion, as well as up‐regulated inflammatory factors including IL‐6, IL‐8 and TNF‐α.^20^ Another study revealed that induced inflammation could also be observed in healthy mice treated with PM2.5.^20^ Treg cells play a regulatory role in the dynamics of Th cells by inhibiting the activation of T cells via IL‐10, thereby maintaining the balance of mature of Th cell maturity in the body.^1^ This mechanism is classic usually seen in microbes' infection. However, the Treg cell‐regulated Th17 cells were activated instead of Th2 cells in the Th2‐low phenotype asthma.^21^ Th‐17‐driven asthma usually exhibits neutrophilic and macrophage inflammation, resulting in a more severe and fatal case when compared to Th2 cells‐driven asthma.^22^ This evidence showed that the inflammatory response triggered by PM2.5 goes in a Th‐17–dependent way rather than Th2 cells.

Secondly, the present study found that the overexpression of miR‐224 could alleviate asthma syndrome of mice by suppressing inflammation and airway remodelling. The altered miRNA profile has been investigated in human asthma samples, thereby confirming the central regulatory role of miRNAs in inflammation.^23^ These miRNAs play regulatory roles in the regulation of cytokines, related receptors or kinases. For example, miR‐155 has been reported to target and regulate IL‐13 generated by Th2 cells.^24^ The miR‐126 has also been verified to be up‐regulated in OVA‐induced asthma mice, which involved in airway inflammation and remodelling.^25^ Furthermore, miR‐26a has been reported to be related with airway remodelling, whereby it could be up‐regulated under mechanical stress and induce human airway smooth muscle hypertrophy by down‐regulating glycogen synthase kinase‐3 beta.^26^ However, we found that low enrichment of miR‐224 in OVA‐asthma mice and the overexpression of miR‐224 could reverse the PM2.5‐driven effects by targeting TLR2. TLR2 is an important receptor in the host immunity and usually recognizes lipopeptides/lipoproteins and wall of gram‐positive bacteria to transduce signals.^27^ Solid particles of PM2.5 usually comprised of the non‐polar molecule that might contribute to activation of inflammation through TLR2. Besides, TLR2 can also recognize allergic antigen through dendritic cells and induce the generation of Th2 cells. Disruption of TLR2 expression ameliorates the airway epithelial inflammatory response to DNA plasmids which exert toxicity in part via unmethylated CpG motifs that stimulate TLR9‐expressing leucocytes.^28^ miR‐224 has been detected to be down‐regulated in nasal mucosa of allergic asthmatics as compared to healthy controls.^29^ More recently, the role of miRNA regulating its target gene has been revealed in numerous immunological and inflammatory disorders, including allergic inflammation. For instance, miR‐21 inhibits TLR2 agonist‐induced lung inflammation in mice.^30^ Decreased miR‐144 could enhance proinflammatory cytokine TNF‐α and IFN‐γ production by targeting TLR2 in vitro, thus eliciting the progression of non‐alcoholic steatohepatitis of high‐fat‐diet‐induced metabolic syndrome E3 rats.^31^ A similar study has reported that, confirmed by in silico prediction analyses, Western blot analysis and luciferase assay, p21 is a specific direct mRNA target of miR‐224 and its expression can be inhibited by miR‐224, thereby accelerating late neoplastic progression in inflammatory bowel disease.^32^ However, the mechanism underlying the down‐regulation of TLR2 by miR‐224 in airway epithelial cells and suppression of inflammatory factors expression as well as MMP9 and TIMP‐1 was unclear, and further investigation is needed. For such a result, we believe that it is related to the characteristics of TLRs. Toll‐like receptor is an important class of protein molecule involved in non‐specific immunity and is also a bridge connecting non‐specific immunity and specific immunity. Toll‐like receptor is a single transmembrane non‐catalytic protein that recognizes molecules with conserved structures derived from microorganisms, and the PM2.5 actually has complex components. Thus, when OVA‐induced asthma mice inhaled PM2.5, physical barriers of the body were triggered, such as skin and mucous membranes. Toll‐like receptors can recognize them and activate the body to produce immune cell responses.

All in all, the present study demonstrated that miR‐224 may act as an anti‐asthma miRNA by suppressing inflammation and airway remodelling in the OVA‐induced asthmatic mice. Our study revealed that miR‐224 targets TLR2 and inhibits Th17 cells‐secreted inflammatory factors, including IL‐4, IL‐5 and IL‐17. Therefore, it is promising to develop therapeutic strategies targeting miR‐224 for clinical use in the treatment of asthma. However, more in‐depth investigations are needed to explore the underlying mechanism of the miR‐224/TLR2 signalling pathway.

## CONFLICT OF INTEREST

None.

## AUTHOR CONTRIBUTIONS

Ping Li and Fengjun Guo designed the study. Jinpeng Wang and Baihong Zheng collated the data, carried out data analyses and produced the initial draft of the manuscript. Xuelei Zhang contributed to drafting the manuscript. All authors have read and approved the final submitted manuscript.
